# Supplementary material for: Hydrodynamic instabilities in membrane systems with current loading, Fourier analysis
Source: PLoS One. 2026 May 4;21(5):e0335005. doi: 10.1371/journal.pone.0335005 (PMC13138678; doi:10.1371/journal.pone.0335005)
Supplement: Attachment — Submitted filename: Paper_Review_anon.pdf [file pone.0335005.s003.pdf]

Reviewer Report

Manuscript Title: Hydrodynamic instabilities in membrane systems with current loading, Fourier analysis
Manuscript ID: PONE-D-25-53895

Summary of the Work

This manuscript investigates the dynamics of transport processes in a horizontal membrane system employing bacterial cellulose (Biofill) membranes. The authors focus on "current loading" conditions, measuring membrane currents in response to electrolyte concentration gradients, rather than the traditional transmembrane voltage measurements. The study compares a stable configuration (Configuration A) against an unstable configuration (Configuration B) across a range of initial concentration quotients. By applying a differential detrending method and Fast Fourier Transform (FFT) to the current time series, the authors characterize the hydrodynamic instabilities arising in the Concentration Boundary Layers (CBLs). A key finding is the non-linear dependence of the instability signal power on the concentration quotient, which exhibits a distinct maximum at a quotient of 2500 before decreasing at higher concentrations.

Specific Comments and Questions for the Authors

While the experimental approach of analyzing current pulsations provides an interesting perspective on hydrodynamic instabilities, I have several concerns regarding the methodology and the physical interpretation of the data that must be addressed:

1. **Differentiation between Convection Types:** The authors suggest that "current loading" introduces additional concentration disturbances compared to voltage measurements. However, the manuscript does not clearly distinguish between hydrodynamic instabilities caused purely by gravitational density gradients (Rayleigh-Bénard type) and those potentially induced or exacerbated by the current flow itself (electro-convection). *Question: Did the authors perform control experiments with varying external resistances or zero-current conditions to isolate the "current load" effect from the density effect?*

2. **Detrending Algorithm and Frequency Response:** The data preparation for FFT involves a differential algorithm defined as $\Delta I(t_i) = I(t_i) - I(t_i + 1\ \text{min})$. This operation effectively acts as a high-pass filter. *Question: How does the specific choice of a 1-minute time lag impact the detection of lower-frequency pulsation modes? Was a sensitivity analysis performed with different time lags (e.g., 2 or 5 minutes) to ensure that the observed peak power at* $C_h/C_l = 2500$ *is not an artifact of the filter width matching a specific process time-scale?*

3. **Statistical Robustness:** The figures currently present single curves for specific concentration quotients. *Question: How many experimental repetitions were conducted for each* C_h/C_l *point in figure? The manuscript should include error bars or standard deviations for the "Average Power" presented in Figure 6 and Figure 10 to demonstrate that the peak*

at 2500 is statistically significant. All rest figures from figure 2 to figure 10 also need error bars.

4. **Physical Mechanism of the Power Decrease:** The results indicate a maximum signal power at $C_h/C_l = 2500$, followed by a decrease at higher quotients (5000, 7500). Classical theory suggests that higher density gradients usually lead to more intense turbulence (higher Rayleigh numbers). *Question: What is the physical mechanism causing the suppression of instability intensity at the highest concentrations? Is this related to a saturation of the membrane transport capacity, a change in solution viscosity, or another hydrodynamic factor?*

5. **Steady-State vs. Dynamic Analysis:** The authors note that for $C_h/C_l > 25$, steady-state currents in Configuration B remain higher than in A due to CBL "blurring." However, for very high concentrations, the difference between steady states appears to decrease. *Question: Does this convergence of steady-state values at high concentrations correlate with the decrease in FFT signal power observed above $C_h/C_l = 2500$? A unified explanation linking the steady-state current data and the FFT dynamic analysis is currently missing.*

Recommendation

I recommend that the authors provide major clarifications regarding the signal processing methods and the physical interpretation of the non-monotonic dependence of signal power on concentration gradients before this work can be accepted.
