# Supplementary material for: Hydrodynamic instabilities in membrane systems with current loading, Fourier analysis
Source: PLoS One. 2026 May 4;21(5):e0335005. doi: 10.1371/journal.pone.0335005 (PMC13138678; doi:10.1371/journal.pone.0335005)
Supplement: Attachment — Submitted filename: Response to Reviewers.docx [file pone.0335005.s004.docx]

**Responses to reviewers' comments**

Manuscript Title: **Hydrodynamic instabilities in membrane systems with current loading, Fourier analysis**

Manuscript ID: **PONE-D-25-53895**

**Review 1:**

1. **Differentiation between Convection Types**: The authors suggest that ”current loading” introduces additional concentration disturbances compared to voltage measurements. However, the manuscript does not clearly distinguish between hydrodynamic instabilities caused purely by gravitational density gradients (Rayleigh-Benard type) and those potentially induced or exacerbated by the current flow itself (electro-convection).

*Question*: Did the authors perform control experiments with varying external resistances or zero-current conditions to isolate the ”current load” effect from the density effect?

AD. 1.

In the case of high externally forced currents (by additional EMF - active current forcing), hydrodynamic instabilities in solutions may occur. However, the case under consideration concerns the passive generation of currents in membrane systems. This means that the currents are generated by differences in electrolyte concentrations at the surfaces of electrodes connected in the membrane system to a nanoammeter (low external resistance) used to measure these currents. Therefore, the values of these currents are not large enough to cause hydrodynamic instabilities on their own. However, they can be used by measuring their changes over time to monitor processes occurring in the membrane system (changes in electrolyte concentrations, formation of CBLs near the membranes, or the appearance and evolution in time of hydrodynamic instabilities related to natural convection in the gravitational field and caused by the density gradients appearing in CBLs opposite to the gravitational field vector). For this reason, the formulations regarding the influence of these currents on hydrodynamic instabilities are too strong, therefore the text of the article was re-examined and such suggestions were removed from the text of the article. Our experiments did not concern determining the influence of the current flowing in the membrane system on hydrodynamic instabilities, but the use of measured current to monitor processes in the membrane system. In the case under consideration, the observed currents are a consequence of changes in concentrations in the membrane system and not the cause of their disturbance. Referring to the reviewer's question, the carried out experiment cannot be considered as allowing the separation of the current effect from the density effect of hydrodynamic instabilities. Additionally, no tests were performed with variable resistances connected in series to the nanoammeter. The idea is interesting, however, due to the passive nature of current generation in the membrane system, it would probably lead to a reduction in current intensities under analogous conditions of the membrane system, without a significant impact on instabilities related to density effects.

2. **Detrending Algorithm and Frequency Response**: The data preparation for FFT involves a differential algorithm defined as ΔI(ti) = I(ti) − I(ti + 1 min). This operation effectively acts as a high-pass filter.

*Question*: How does the specific choice of a 1-minute time lag impact the detection of lower-frequency pulsation modes? Was a sensitivity analysis performed with different time lags (e.g., 2 or 5 minutes) to ensure that the observed peak power at Ch/Cl = 2500 is not an artifact of the filter width matching a specific process time-scale?

AD. 2.

The selection of the time difference (Δt=1min) was preceded by an analysis of the behavior of the differential signal for various time differences. The new differential signals allowed the decreasing trend to be eliminated, but after transformations remained pulsations of various amplitudes and this same frequencies. Due to the analysis of current signals (their changes over time) in the tested range of initial conditions of the membrane system (pulsation periods were greater than 1 minute), the signal shift time in the differential algorithm was chosen to be 1 minute. The analysis of selected signals with different shift times (up to 5 minutes) shows that this does not disturb the signal pulsations (i.e. their frequency character) but only affects their amplitudes. Therefore, the main criterion for selecting the shift time were the observed pulsations (their frequencies) of the recorded membrane currents. As can be seen from the Fourier analysis, the main frequencies of the membrane currents detected in the spectrum are in the range of values below 1 min^-1^ (main band between 0.05 and 0.06 min^-1^). The example graphs after applying a 2-minute and 5-minute shift (compared to 1 minute), (for configuration B and (Ch/Cl)_o_ =2500) are shown in the figure on the next page. (Perhaps using greater signal shift times could result in "clipping" of the parts of signal at higher frequencies – around 0.5 min^-1^, occurring in signals with higher (Ch/Cl)_o_ values).

3. **Statistical Robustness**: The figures currently present single curves for specific concentration quotients. Question: How many experimental repetitions were conducted for each Ch/Cl point in figure? The manuscript should include error bars or standard deviations for the ”Average Power” presented in Figure 6 and Figure 10 to demonstrate that the peak

1 at 2500 is statistically significant. All rest figures from figure 2 to figure 10 also need error bars.

AD. 3.

The experiment was aimed at measuring a series of measurements for different initial conditions (different (Ch/Cl)_o_). Measurements performed for the same conditions (both for voltages between electrodes and currents flowing in the membrane system) show that the standard error of measurements of the initial current do not exceeded 5%, while the standard error of measurements of currents in steady states do not exceeded 12%. The estimations of the measurement error of the current intensities in the initial and in the steady states were performed five times for the selected initial quotients of NaCl concentrations in the chambers (*C*_h_/*C*_l_)_o_. An error of this order can be plotted on the graphs of initial and steady-state states in configurations A and B as a function of (Ch/Cl)_o_ (Fig. 3). As for the curves (Power as a function of (Ch/Cl)_o_- Figs. 6 and 10), the calculations were performed for sample single I(t) runs and therefore the measurement accuracy cannot be taken into account in these graphs.

a

b

c

**Fig. R1** Time characteristics of differences between elements of the series of current intensities (Δ*I*) for configuration B of the membrane system and for the initial NaCl concentrations quotients *C*_h_/*C*_l_ = 2500 on the membrane, and for Δ*t* equal to: 1min (a), 2min (b) and 5min (c).

4. **Physical Mechanism of the Power Decrease**: The results indicate a maximum signal power at Ch/Cl = 2500, followed by a decrease at higher quotients (5000, 7500). Classical theory suggests that higher density gradients usually lead to more intense turbulence (higher Rayleigh numbers). Question: What is the physical mechanism causing the suppression of instability intensity at the highest concentrations? Is this related to a saturation of the membrane transport capacity, a change in solution viscosity, or another hydrodynamic factor?

AD. 4.

In the observed hydrodynamic instabilities, increasing the initial concentration gradients through the membrane causes larger density gradients to appear in the CBLs over time, resulting in more intense hydrodynamic instabilities in configuration B of the membrane system. However, when measuring the temporal changes in membrane voltages for various membranes, we observed large voltage pulsations for (Ch/Cl)_o_ greater than or equal to 5000, which were strongly damped after some time of appearance. As a result of hydrodynamic instabilities, intense stirring of solutions in the chambers occurs in an area larger than the CBLs. Increase of the initial concentration gradient across the membrane (increase of (Ch/Cl)_o_) causes greater electrolyte transport through the membrane, resulting in a faster increase of concentration gradients (and solution density) in CBLs and higher intensities of hydrodynamic instabilities (pulsations of both observed voltages and currents with higher amplitude and frequency). However, at high (Ch/Cl)_o_ values (as was noted by the reviewer), there may be a saturation effect of electrolyte transport through the membrane. Besides, a faster equalization of concentrations in the chambers (at the level of higher concentrations) may occur. These effects may finally lead to a faster decrease of concentration gradients (as well as density gradients) in the chambers which may lead to faster damping of instability (and thus pulsation of observed electrical parameters). Previous analyses of concentration changes at membrane surfaces in time for the stable configuration after turning off mechanical stirring of solutions indicate that the largest changes in these concentrations occur within a few minutes after turning off mechanical stirring. After this time, membrane transport is significantly limited by the formation of CBLs. The emerging hydrodynamic instabilities in CBLs caused by gravity also disturb the concentrations at the membrane surfaces, leading to temporary increase in membrane transport, or rather, pulsatile changes in these concentrations. This is due to the opposing effects of diffusion through the membrane and hydrodynamic instabilities on changes in concentration at the membrane surfaces. Perhaps, for large (Ch/Cl)_o_, these relative concentration disturbances at the membrane are smaller compared to smaller (Ch/Cl)_o_, which may result in a lower possibilities of reproducing density gradients leveled by hydrodynamic instabilities. As a result, a damping effect on hydrodynamic instabilities sustained by density gradients in the chambers solutions can be observed. Due to the fact that the signal power in the Fourier transform is connected with the pulsation intensity, it can be stated that a greater damping of current pulsations over time causes a decrease in the average power in the considered frequency range. In the case of the twenty-minute interval with the interval centered at the 20th minute, due to the high intensity of pulsation in the initial phase of instability, high average powers in this time interval are observed also for (Ch/Cl)_o_ greater than (or equal to) 5000. These processes and their potential influence on the nature of instability (current pulsations) were taken into account in explaining the dependence of the average power as a function of (Ch/Cl)_o_.

5. **Steady-State vs. Dynamic Analysis**: The authors note that for Ch/Cl > 25, steady state currents in Configuration B remain higher than in A due to CBL ”blurring.” However, for very high concentrations, the difference between steady states appears to decrease. Question: Does this convergence of steady-state values at high concentrations correlate with the decrease in FFT signal power observed above Ch/Cl = 2500? A unified explanation linking the steady-state current data and the FFT dynamic analysis is currently missing.

AD. 5.

Indeed, as was noted by the reviewer, the difference between the steady states for configurations A and B of the membrane system – shown in Fig. 3 for large (Ch/Cl)_o_ (above 750) decreases compared to smaller (Ch/Cl)_o_ values (between 100 and 750). The likely cause is (similarly to the observed pulsation intensities and the associated average signal powers obtained in Fourier analysis) the effect of damping the instability over time. It can be suspected that the time after which the instabilities are suppressed due to the effective disappearance of density (concentration) gradients is shorter for higher (Ch/Cl)_o_, and during measurements up to 300 minutes it is visible for (Ch/Cl)_o_ greater than (or equal to) 5000. It is true that for smaller (Ch/Cl)_o_ smaller amplitudes of hydrodynamic instabilities are observed (smaller amplitudes and frequencies of current or voltage pulsations), but due to their longer duration they may result in larger differences between the steady states for configurations A and B compared to larger (Ch/Cl)_o_.

**Review 2:**

**Abstract**

The abstract is overly descriptive and lacks quantitative clarity. Trends are reported but key numerical outcomes or uncertainty estimates are missing, making it difficult to assess the strength of the findings. Terminology such as “initial concentration quotient on the membrane” is used repeatedly without clearly defining whether this refers to bulk concentrations or boundary-layer concentrations.

The abstract does not mention experimental replicates or variability, which is important for technical assessment.

Ad. (Abstract):

Analyzing the hydrodynamic instabilities in configuration B of the membrane system, by means of the Fast Fourier Transform in the range of time from 50 to 250 min. we can state that the average power of the current signal nonlinearly depends on the (Ch/Cl)_o_ (initial bulk solutions in chambers, homogeneous during mechanical stirring of solutions) with maximum at (Ch/Cl)_o_ = 2500. The transformation of current signal (difference of currents with shift time equal 1 minute) allows to eliminate the decreasing trend line connected with rebuilding of CBLs near membrane. The Short Time Fourier Transform (STFT) of transformed current signal with 20 minute rectangular windows shows increasing amplitudes with increasing (Ch/Cl)_o_ up to 2500 (in later times and higher frequencies). For (Ch/Cl)_o_ greater than 2500 lower amplitudes of transformed signals for higher frequency and longer times were observed, what can be associated with higher dumping of hydrodynamic instabilities in the membrane system. Calculation of average power in 20min intervals showed similar dependencies of average power as a function of (Ch/Cl)_o_ in all intervals, as was the case for the signal in the range of time from 50 to 250 min. The abstract and methodology have been revised.

**Introduction**

The introduction provides extensive theoretical background but does not clearly define the specific unresolved technical question addressed by this study. Literature positioning is limited, with strong reliance on prior work from the same research group and limited comparison with broader recent literature. The aim statement is descriptive rather than hypothesis-driven, which reduces clarity regarding the study’s objectives.

Ad. (Introduction):

In the introduction, we have improved the description of the article's objectives and extended the signal analysis based on the Short Time Fourier Transform (STFT) method. The Fast Fourier Transform (FFT) and Short Time Fourier Transform (STFT) of the current signal and its "differential" transformation were used to demonstrate the complex nature of the distribution of signal power maxima and amplitude distribution. The Fourier transform of membrane currents could indicate the random (stochastic) nature of hydrodynamic instabilities related to natural convection, induced by sufficiently large solution density gradients near the membrane surface.

**Materials and Methods**

The manuscript does not specify the number of independent experiments, whether figures represent single runs or averages, or the variability between experiments. This limits reproducibility. No statistical analysis or uncertainty propagation is described for FFT-derived parameters or current measurements. The FFT methodology is not fully defined. Important details such as windowing, normalization, detrending strategy, and handling of spectral leakage are missing. The difference method (ΔI) is introduced later in the Results section rather than being fully described in Methods, which affects reproducibility. The potential influence of instrument loading on the measurements is acknowledged but not quantitatively evaluated.

Ad. (Materials and Methods):

The measurements were performed as a series for various (Ch/Cl)_o_. Independent measurements showed that the standard error of measurements of the initial current do not exceeded 5%, while the standard error of measurements of currents in steady states do not exceeded 12%. The estimations of the measurement error of the current intensities in the initial and in the steady states were performed five times for the selected initial quotients of NaCl concentrations in the chambers (*C*_h_/*C*_l_)_o_.

We have moved (and improved) the description of the signal differentiation algorithm from "Results and Discussion" “into the methodology”:

<<

Because we focus on the analysis of hydrodynamic instabilities, in order to eliminate the systematic decrease of membrane currents resulting from the first process, the differential method was used. This method consists in replacing the points of the measurement series with the differences between the points of the time series that are separated in time by the same interval. We used the algorithm: Δ*I*(*t*_i_) = *I*(*t*_i_) – *I*(*t*_i+1min._). The time interval between the points amounts one minute because no pulsations with a period of less than 1 minute are observed. For smaller values of the time interval, the graphs are similar but with a smaller amplitude and a larger number of high-frequency components not observed in the current signal.

>>

We have clarified the parameters of the Fourier transform.

Detailed characteristics of the FFT and STFT methodologies:

Fast Fourier Transform (FFT) ( frequency power spectrum of current signal) :

analyzed time interval – from 50 min. to 250 min. and in twenty-minute intervals;

sampling – every 4 s;

average power in the 50-250 min. interval and in twenty-minute intervals.

Short Time Fourier Transform (STFT): (amplitude distribution on the plane: time – frequency)

window type – rectangular;

sampling – every 4 seconds,

number of samples – 301 (20-minute intervals);

parameter – amplitude;

Additionally, in the graphs below we have presented comparative results of the STFT transformation (for (Ch/Cl)o = 2500) for selected parameters of transformation.

**Fig. R2** Exemplary comparisons of alternative possibilities for different transformation (STFT) parameters for (Ch/Cl)_o_ = **2500**:

• Sampling interval 4s = 0.0666(7)min ; FFT Length: 512 ; Window Length: 301 (20min window) ; Overlap: 150 ; Window correction: Amplitude ; Option: Amplitude result;

Window type **rectangle**	↔	Window type **Gaussian**

 Origin Pro windows

• Sampling interval 4s = 0.0666(7)min ; Window Length 301 (20min window) ; Overlap 150 ; Window type rectangle; Window correction Amplitude ; Option Amplitude result;

FFT Length **512**	↔	FFT Length **1024**

• Sampling interval 4s = 0.0666(7)min ; FFT Length 512; Window Length 301 (20min window) ; Overlap 150 ; Window type rectangle; Option Amplitude result;

Window correction **Amplitude**	↔	Window correction **Power**

• Sampling interval 4s = 0.0666(7)min ; FFT Length 512; Overlap 150 ; Window type rectangle; Window correction Amplitude; Option Amplitude result;

Window Length **301** (**20min** window)	↔	Window Length **512** (**34min** window)

• Sampling interval 4s = 0.0666(7)min ; FFT Length 512; Overlap 150 ; Window type rectangle; Window correction Amplitude; Option Amplitude result;

Window Length **301** (**20min** window)	↔	Window Length **150** (**10min** window)

**Results and Discussion**

Results and interpretation are strongly mixed, making it difficult to distinguish direct observations from mechanistic interpretation.

No uncertainty estimates or error bars are presented in figures supporting key conclusions, including the reported maximum signal power at Ch/Cl = 2500. Mechanistic explanations of hydrodynamic instability are inferred from signal behavior without direct validation through flow visualization or complementary measurements. FFT interpretation appears overextended, as frequency shifts are discussed mechanistically without a quantitative supporting model. There are figure referencing inconsistencies in the text, suggesting editing issues.

Ad. (Results and discussion):

The structure of the results and discussions has been carefully rethought and improved.

From the new Figure 9 it results that the STFT of the current signal after elimination of the decreasing trend shows a dependence of the distribution of areas with higher amplitudes in the time-frequency space on (Ch/Cl)_o_. The increase of (Ch/Cl)_o_ causes these areas to expand (with increasing amplitude values) towards higher frequencies and longer observation times. This is due to the fact that after eliminating the trend line, the graphs obtained as a result of STFT for “differences of currents” better characterize the observed current pulsations and the related hydrodynamic instabilities than the STFT characterization of the current signal itself. The expansion of the regions with larger amplitudes in space ( time - frequency ) can be associated with the increasing intensity of instabilities in chambers of the membrane system, characterized by higher frequencies of solution movements due to gravitational convection caused by solution density gradients. At the same time, with the increase of (Ch/Cl)_o_ (in the range up to 2500), the increasing intensity of instabilities (larger amplitudes of STFT) last longer (also at (Ch/Cl)_o_ = 2500) and do not disappear in the last interval of time. This indicate the durations of hydrodynamic instabilities (for (Ch/Cl)_o_ > 100 ) longer than 300 minutes after switching off mechanical stirring of solutions (ensuring the homogeneity of solutions in the chambers of the membrane system at the initial moment). Further increase of (Ch/Cl)_o_ (above 2500) causes the areas of high amplitudes to be significantly reduced for higher frequency and time ranges. Large amplitudes for times below 50 minutes and over a wide frequency range decay rapidly with increasing observation time. This behavior of current pulsations can be attributed to the initially high intensity of hydrodynamic instabilities (in the first 50 minutes), which as expected, lead to intense stirring of solutions in the chambers. High intensity of solutions stirring associated with hydrodynamic instabilities results in faster equalization of solutions densities and thus the disappearance of density gradients in solutions. With the limited rate of CBLs rebuilding due to membrane transport at relatively higher concentrations of solutions at the membrane surfaces (compared to lower (Ch/Cl)_o_), the effect of rapid suppression of hydrodynamic instabilities may appear. As can be seen from new Figures 6e and 6f, for (Ch/Cl)_o_ = 7500 the region of large amplitudes is wider (in frequency space) in the initial 50 minutes, but it decays much faster compared to the case of (Ch/Cl)_o_ = 5000.

**Figures and Figure Legends**

Figure legends are not fully self-contained; several lack information about sample size, preprocessing steps, or units for FFT power.

The large number of multi-panel FFT figures reduces clarity; summary metrics could improve readability.

Variability or reproducibility across experiments is not shown in any figure.

Ad. (Figures and Figure Legends):

Figure descriptions have been corrected according to the reviewer's suggestions.

In the measurements, after the initial preparation and checking of the system (and determination of the measurement accuracy), a series of measurements was performed for specific configurations of the membrane system (A and B configurations with membrane in horizontal location) and selected (Ch/Cl)_o_ (initial concentrations of solutions in the chambers – bulk solutions).

The units in the parameters used have been checked.

The graphs with additional panels showing selected frequency ranges in detail for better visualization have been rethought and in cases where they could be omitted, the panels have been removed.

Measurement errors are included in the appropriate graphs.

**Conclusions**

The conclusions mainly restate observations rather than demonstrating validated mechanisms. Some conclusions appear stronger than what is directly supported by the presented data. Limitations of the study are not discussed, particularly the absence of flow visualization, lack of statistical reporting, and potential measurement-system effects.

Ad. (Figures and Figure Legends):

The conclusions have been revised (and shortened to essential ones) in accordance with the reviewer's suggestions. In the introduction and conclusions, we attempted to address the limitations resulting from the lack of extended research to include visualization of hydrodynamic instabilities. We did not perform parallel visualization studies due to the different structures and instrumentation of the membrane systems, but we used the experience of other authors who published such considerations (not only for membrane systems but also for others in which hydrodynamic instabilities could occur).

The distribution of peaks in the FFT spectrum of the current signal after transformation indicates the random nature of the analyzed hydrodynamic instabilities in the membrane system.
