# Supplementary material for: Hydrodynamic instabilities in membrane systems with current loading, Fourier analysis
Source: PLoS One. 2026 May 4;21(5):e0335005. doi: 10.1371/journal.pone.0335005 (PMC13138678; doi:10.1371/journal.pone.0335005)
Supplement: Attachment — Submitted filename: reviewer comments.docx [file pone.0335005.s002.docx]

**Hydrodynamic instabilities in membrane systems with current loading, Fourier analysis**

**Comments for authors**

The manuscript investigates hydrodynamic instabilities in membrane systems using current measurements and Fourier analysis. The topic fits the technical scope of PLOS ONE and the experiments appear carefully conducted. However, several technical and reporting weaknesses reduce the clarity and reproducibility of the study and should be addressed before the manuscript can be considered technically sound.

**Abstract**

The abstract is overly descriptive and lacks quantitative clarity. Trends are reported but key numerical outcomes or uncertainty estimates are missing, making it difficult to assess the strength of the findings.

Terminology such as “initial concentration quotient on the membrane” is used repeatedly without clearly defining whether this refers to bulk concentrations or boundary-layer concentrations.

The abstract does not mention experimental replicates or variability, which is important for technical assessment.

**Introduction**

The introduction provides extensive theoretical background but does not clearly define the specific unresolved technical question addressed by this study.

Literature positioning is limited, with strong reliance on prior work from the same research group and limited comparison with broader recent literature.

The aim statement is descriptive rather than hypothesis-driven, which reduces clarity regarding the study’s objectives.

**Materials and Methods**

The manuscript does not specify the number of independent experiments, whether figures represent single runs or averages, or the variability between experiments. This limits reproducibility.

No statistical analysis or uncertainty propagation is described for FFT-derived parameters or current measurements.

The FFT methodology is not fully defined. Important details such as windowing, normalization, detrending strategy, and handling of spectral leakage are missing.

The difference method (ΔI) is introduced later in the Results section rather than being fully described in Methods, which affects reproducibility.

The potential influence of instrument loading on the measurements is acknowledged but not quantitatively evaluated.

**Results and Discussion**

Results and interpretation are strongly mixed, making it difficult to distinguish direct observations from mechanistic interpretation.

No uncertainty estimates or error bars are presented in figures supporting key conclusions, including the reported maximum signal power at Ch/Cl = 2500.

Mechanistic explanations of hydrodynamic instability are inferred from signal behavior without direct validation through flow visualization or complementary measurements.

FFT interpretation appears overextended, as frequency shifts are discussed mechanistically without a quantitative supporting model.

There are figure referencing inconsistencies in the text, suggesting editing issues.

**Figures and Figure Legends**

Figure legends are not fully self-contained; several lack information about sample size, preprocessing steps, or units for FFT power.

The large number of multi-panel FFT figures reduces clarity; summary metrics could improve readability.

Variability or reproducibility across experiments is not shown in any figure.

**Conclusions**

The conclusions mainly restate observations rather than demonstrating validated mechanisms.

Some conclusions appear stronger than what is directly supported by the presented data.

Limitations of the study are not discussed, particularly the absence of flow visualization, lack of statistical reporting, and potential measurement-system effects.
